# Persistent postural-perceptual dizziness: a useful new syndrome

**DOI:** 10.1136/practneurol-2017-001817

**Published:** 2018-01-04

**Authors:** Barry M Seemungal, Luca Passamonti

**Affiliations:** 1 Division of Brain Sciences, Imperial College, London, UK; 2 Department of Clinical Neurosciences, University of Cambridge, Cambridge, UK

**Keywords:** PPPD, vertigo, chronic dizziness, functional neurological disorder, vestibular therapy

In this issue of *Practical Neurology*, Popkirov, Staab and Stone illuminate a newly defined condition—persistent postural-perceptual dizziness or ‘PPPD’,^1^ ^2^ a maladaptive functional syndrome in which patients feel unbalanced despite not falling, and feel that they are moving, despite being stationary. PPPD is common in specialist dizzy clinics, accounting for 10% of cases as a primary diagnosis of dizziness.^3^ PPPD can coexist with other causes of dizziness, such as vestibular migraine or benign paroxysmal positional vertigo, and it is in this form that it most commonly presents to a specialist dizzy clinic.

PPPD is a complex functional neurovestibular disorder thought to arise from the ‘mismatch’ between ‘bottom-up’ inputs (ie, vestibular and/or proprioceptive) and maladaptive signals from ‘top-down’ attentional control systems (ie, anxiety-driven hypervigilance). Current models show that the brain acts as a Bayesian estimator in which both prior estimates (‘beliefs’) and current information influence the state of sensory and motor processes. 4 Popkirov, Staab and Stone’s proposal for the pathophysiological model of PPPD invokes a disturbance in the brain’s Bayesian estimator, such that PPPD and functional neurological disorders in general are driven by excessively precise top-down *a priori* beliefs.^4^ However, at present, this only remains an intriguing hypothesis that requires additional testing and empirical evidence.

For PPPD, important internal estimates might include those of body motion and body orientation with respect to gravity (with attendant impacts on motor control of gait and balance). For example, when looking out of the window while seated in a stationary train, seeing an adjacent moving train may trigger a momentary sensation of self-motion. The perceptual consequences of visual–vestibular interaction follow Bayesian rules. In the train illusion, therefore, prior experience indicates that wide-field visual motion typically arises from our own motion. This is because the probability of a large object, such as a tree, moving on its own is close to zero.

The other prominent aspect of PPPD is a sense of imbalance and occasionally a functional gait disorder. Human upright walking is a complex and species-defining function; it takes infants almost 2 years to learn its rudiments, similar to some higher order cognitive functions. Bipedal walking is inherently unstable, requiring us to move our centre of mass in front before every step; in effect, every human step is to nearly fall, avoided only by a complex whole-body coordination. Human infants show a daredevil determination, characterised by impulsivity and recklessness, to learn to walk, and despite repeated falls do not develop a fear of falling. Hence, learning to walk requires an appropriate balance between reward and fear circuits. In adulthood, falling could be calamitous; thus, situations that challenge our balance (or at least are perceived as such) activate fear circuits, particularly in the elderly. During fearful walking, for example walking on an open elevated platform, we are very attentive and avoid moving the body’s centre of mass outside of our stance, with slow, low-amplitude leg movements maintaining close contact to the ground and a rigid upper body. Such a gait develops in some people with PPPD.

Patients with PPPD often show context-dependent aggravation of their symptoms, typically avoiding shopping malls and supermarkets—often misinterpreted as agoraphobia. These visually busy environments challenge their internal estimates of bodily stationarity or self-motion. Some patients with PPPD have a sense of instability and manifest a fearful gait response when walking on smooth or open surfaces. Occasionally PPPD—in a syndrome previously called motorists’ vestibular disorientation syndrome^5^—can affect driving with a distorted sense of vehicular tilt when driving on open roads, and with a very specific speed threshold for developing symptoms. Patients often change multiple cars before seeking medical help.

It is unclear why some patients develop PPPD after a trigger such as a vestibular disorder, and others do not. Subjects’ idiosyncratic increased use of visual cues for balancing may be at the core of PPPD.^6^ Neuroticism and introversion are also linked with PPPD but not with other non-PPPD disorders that might have a comparable level of disability (eg, vestibular neuritis).^7^ ^8^ Furthermore, personality traits such as neuroticism in healthy people influence key brain visuovestibular and anxiety networks that may be at the basis of PPPD.^9^ ^10^ In the model outlined by Popkirov, Staab and Stone (figure 2), neuroticism and introversion increase the risk of developing PPPD, which in turn interacts with provoking elements (eg, vestibular migraine) and aggravating factors (eg, upright posture) to perpetuate the ‘vicious’ circle of maladaptation that ultimately leads to PPPD. Although psychiatric comorbidity is common in PPPD, it is neither necessary or sufficient to cause PPPD. In keeping with this notion, the brain changes detected in visuovestibular brain networks in PPPD are independent from psychiatric comorbidity and personality differences.^11^ ^12^

Popkirov, Staab and Stone also indicate that PPPD may coexist with other diagnoses that cause dizziness. We emphasise that if there are coexisting balance disorders (eg, Benign Paroxysmal Positional  Vertigo, vestibular migraine, vestibular neuritis, Ménière’s disease, central neurological conditions), these accompanying disorders should be managed either first or in parallel to PPPD therapy. Migraine deserves a special mention since in patients with active migraine, clinical observation suggests that vestibular rehabilitation may trigger or aggravate migraine and/or PPPD symptoms, leading to failure of vestibular therapy. Some vestibular neurologists therefore recommend having a low threshold for using prophylactic agents to treat migraine in patients requiring vestibular therapy, even if migraine is not the primary cause of the vestibular disturbance.

Although vestibular exercises can help to tackle PPPD, combining them with psychotherapies and other approaches that target anxiety-related symptoms and lifestyle-associated disorders—for example, good sleep hygiene, physical exercise, and a balanced diet—may be ideal to accelerate recovery from the maladaptive cycle that perpetuates PPPD. Future research should also include the effect of potentially enjoyable vestibular stimulation in PPPD, such as that derived from sports like tennis or dancing, which are known to mediate brain plasticity within vestibular–cerebellar circuits.^13^

Nevertheless, some important issues remain before we can employ such strategies, including the key pathophysiological mechanisms that underly PPPD and establishing the potential of neuroimaging and other biomarkers for tracking the development and evolution of PPPD. Popkirov, Staab and Stone’s proposal for a new theoretical account to conceptualise PPPD lays the groundwork to move in this direction.
